# COVID-19 and OECD Labour Markets: What Impact on Gender Gaps?

**DOI:** 10.1007/s10272-021-0993-6

**Published:** 2021-10-05

**Authors:** Monika Queisser

**Affiliations:** ELS Social Policy Division, OECD, 2 Rue André Pascal, 75775 Paris Cedex 16, France

## Abstract

Across the board statements on who suffers most are not helpful and may actually be a disservice to the fight for greater gender equality.

The COVID-19 pandemic has been creating a profound economic, social and health shock worldwide, with different implications for men and women.

Around the world, women continue to be at the forefront of the battle against the pandemic (OECD, 2020c). Women make up two-thirds of the health workforce worldwide, for instance, including 85% of nurses and midwives (Boniol et al., 2019); across OECD countries, they also account for 90% of long-term care workers (OECD, 2020b). This exposes women to greater risk of infection; at the same time, women are under-represented in leadership in the health care sector, and often lack a seat at the table when decisions are being made.

Evidence from past economic and health crises suggests that shocks on the scale of the COVID-19 pandemic often impact men and women differently (Alon et al., 2020; Rubery and Rafferty, 2013). The 2008 financial crisis, for instance, was characterised by greater job losses in male-dominated sectors (notably construction and manufacturing) and an increase in hours worked by women, especially in the early years (Sahin et al., 2010; OECD, 2012). During the recovery phase, men's employment improved more quickly than that of women (Périvier, 2014).

There are very good reasons to be concerned about the impact of the pandemic on women's jobs. But is it fair to say that women are bearing the biggest labour market losses in this crisis? To address gender inequalities systematically, it is important to take a closer look at the data. Across the board statements on who suffers most are not helpful and may actually do a disservice to the fight for greater gender equality.

* The author thanks Willem Adema, Jonas Fluchtmann and Valentina Patrini, all from the OECD Social Policy Division, for their inputs and comments.

**Monika Queisser**, Organisation for Economic Co-operation and Development, Paris, France.

## How has women's employment been affected by COVID-19?

Many of the industries that have been most obviously affected by the crisis – e.g. those that rely on physical customer interaction – are also major employers of women. On average across OECD countries, women make up about 53% of employment in food and beverage services (e.g. cafés, restaurants and catering) and 60% in accommodation services (e.g. hotels). In the retail sector, on average, 62% of workers are women, and accounting for a share of three quarters or more in Latvia, Lithuania and Poland.

But when we look at the hard numbers – those from national labour force surveys, for example – the picture becomes more nuanced. Key indicators are not only employment and unemployment rates, but also labour force participation. Many people who lost their jobs are considered inactive in the statistics rather than unemployed, since they were not actively looking or available for paid work; this may be due to an increased burden of unpaid work during the confinement or because lockdowns made job searches difficult. As a consequence, the number of people considered not part of the labour force has risen in all countries. This, in turn, translates into a fall in the labour force participation rate.

As has been pointed out by many, female *labour force participation* has indeed declined, erasing some of the gains made in previous decades (Figure 1, panel a). The labour force participation of women aged 15-64 years (seasonally adjusted) in the OECD area had fallen by 1.16 percentage points in Q1 2021 relative to Q4 2019, slightly more than the labour force participation of men (1.10 percentage points).

Turning to percentage point changes in employment rates shows on average that women's employment (-2.00 percentage points) was impacted slightly less than that of men (-2.18 percentage points). However, in terms of relative changes (not shown here) and given higher male employment overall, women's employment (- 3.3%) dropped somewhat more than men's (-2.9%). Figure 1, panel b, shows a larger drop in women's employment rate compared to that of men in Belgium, Colombia, Latvia and Slovenia, as well as in Canada, Lithuania and Sweden, and to a lesser extent also in Chile, Denmark, Finland and Estonia. In Australia, France, Hungary, Luxembourg, the

Figure 1

**Changes in labour force participation, employment rate and unemployment rate since the onset of the COVID-19 pandemic**

in percentage points

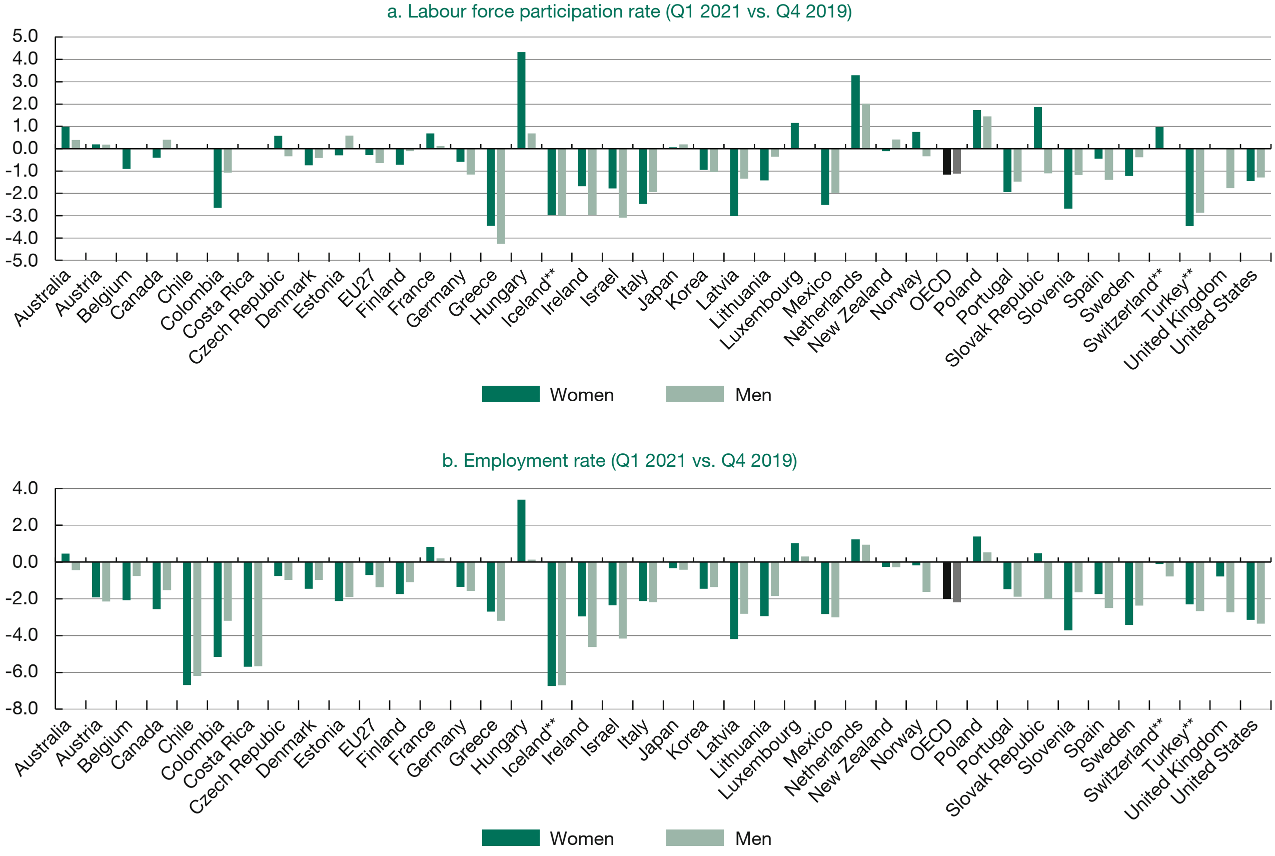

Note: * Data for Iceland, Switzerland and Turkey refer to the difference in the employment rate between Q4 2019 and Q4 2020. No data is provided for Chile and Costa Rica. ** Data for Iceland, Switzerland and Turkey refer to the difference in the employment rate between Q4 2019 and Q4 2020.

Source: OECD, Short-term Labour Market Database, 2021.

Netherlands, Poland and the Slovak Republic, in contrast, women's employment rates actually increased since the onset of the COVID-19 pandemic, while in other countries, such as Ireland, Israel, Norway, Spain, Switzerland and the United Kingdom, men's employment rate saw a stronger decline than that of women. In most countries, however, gender differences in the changes of employment rates were only small. In the European Union overall, the gender employment gap has actually decreased slightly (from 10.7 percentage points in Q4 2019 to 10 percentage points in Q1 2021).

Looking at *unemployment* rates, it is true that women were more affected at the start of the pandemic. As a consequence, the gender unemployment gap increased sharply from March to April 2020 (from 0.3 to 0.9 percentage points), but since then, the gender gap has been narrowing steadily. Between March 2020 and June 2021, the women's unemployment rate actually decreased in Australia, Canada, France and Korea, while the increase in male unemployment was stronger in Austria, Estonia, Finland, Mexico, Sweden and the United States. In June 2021, across the OECD, the gender gap in the unemployment rate slightly increased from just before the pandemic. It stood at 0.4 percentage points in June 2021 compared to 0.3 percentage points in March 2020.

It is important to note, however, that in many countries, especially in Europe, the full impact of the crisis on jobs for both men and women still remains to be seen. The massive use of job retention schemes means that employment has so far fallen only modestly in some countries, and not at all in others. Uncertainty is due to the possibility of further waves of new COVID-19 variants, as well as the economic impact of the various policy measures taken by countries and the gradual phasing out of public support measures. The gender gaps in the labour market will be shaped by questions regarding which sectors will be most affected, how restrictions on childcare and schools affect parents' labour force participation, and how companies adapt their work practices and introduce new technologies.

### What happened to unpaid work?

The pandemic has also increased the burden of unpaid work. Increased caregiving can have an immediate impact as caregivers – typically women – might drop out of the labour market to take care of children and elderly relatives. But there is also a longer-term negative impact on gender equality. As caregivers, i.e. mostly women, reduce their working hours or switch to jobs which may have more flexibility and shorter commuting times, they may also limit their potential of increasing their earnings over time. This is due to a more limited pool of jobs meeting these criteria, possibly weaker bargaining power and fewer opportunities for career development compared to full-time workers. These effects of the pandemic might become apparent over the longer term and be felt for many years (OECD, 2021c).

Even before the pandemic, unpaid work was disproportionately taken on by women. There is not a single OECD country in which men do the same or more unpaid work as women. Across the OECD on average, at just over four hours per day, women systematically spend around two hours per day more on unpaid work than men (OECD, n.d.; OECD, 2021a). Gender gaps in unpaid work are largest in Japan and Korea (2.5 hours) and Turkey (four hours per day), where traditional norms on gender roles prevail. However, even in Denmark, Norway and Sweden – countries that express strong and progressive attitudes towards gender equality – gender gaps in unpaid work still amount to about one hour per day.

Due to the COVID-19 crisis, according to early evidence, women have often been taking on much of the additional unpaid work caused by school and child care closures. And this has been happening even when both parents were confined to the home and a more equal distribution of additional care and non-care household work would have been possible.

In Italy and Spain, for example, most of the additional unpaid work burden fell on women, even though men increased their contribution too (Del Boca et al., 2020; Farré et al., 2020). The increased workload, in particular non-care housework, such as cooking, cleaning and shopping, disproportionately increased relative to that of men. Unpaid house work also increased in the United Kingdom and the Unites States, but evidence as to whether that led to more egalitarian sharing is mixed (Carlson et al., 2020; Hupkau and Petrongolo, 2020; Oreffice and Quintana-Domeque, 2020; Xue and McMunn, 2021). Sevilla and Smith (2020) find that the gender balance in additional childcare provided by men depended on whether men were working from home and whether they had lost their job or been furloughed.

Even when the additional housework burden is on average equally shared between men and women, the pandemic may affect views on how housework is shared within their own households as well as people's opinions about how it is shared across the population. For example, in Germany, the housework burden increased by about an hour for each household and was equally shared between men and women (Zinn, 2020; Hipp and Bünning, 2020; Kreyenfeld and Zinn, 2021). However, because of a substantial imbalance in the distribution of housework to the disadvantage of women prior to the pandemic, most German women judged the overall division as unfair, while two-thirds of German men were of the opposite opinion (Bertelsmann Stiftung, 2020). At the same time, in Germany, the pandemic has moved some households towards extremes in gender housework distribution, meaning that either women take on almost all unpaid housework or that there is a role reversal where men take on the majority of unpaid housework (Hank and Steinbach, 2020). Despite the increased burden for women in many countries, men generally increased their housework contribution during the pandemic, albeit from an often low base. In the long term, this may shift gender norms around unpaid work as men have become more exposed to the burden of domestic work and primary caregiving (Alon et al., 2020; Hupkau and Petrongolo, 2020).

### What policies will help change the gender balance?

Some groups of women have been mastering the crisis comparatively well while others were already struggling before the crisis and even more so during the pandemic. Frontline workers, single parents, part-time workers in precarious jobs – all of these predominantly female groups faced particular challenges and pressures. Self-employed women belong to another at-risk group in the most affected sectors. But what makes them vulnerable above all is their socioeconomic and work situation, which is determined by structural problems rather than the fact that they are women.

The policies needed to reduce risks of increasing gender inequalities and support the labour market participation of women, both in general and for vulnerable groups during the pandemic, are not fundamentally different from those needed before the pandemic.

### Affordable quality childcare is more important than ever

For example, the pandemic has provided further proof of how important it is for working parents to be able to access affordable quality childcare. During the pandemic, many OECD countries assisted parents by providing additional paid or unpaid leave, cash benefits and emergency childcare facilities for essential service workers. But in countries where childcare is more often privately rather than publicly provided, such as Australia and the United States, many childcare facilities closed and there is now a supply shortage (The New York Times, 2021; Jackson, 2021).

It is therefore important to tackle more than just the short-term childcare needs during the crisis and the closure of childcare facilities. Structural barriers such as the high cost of non-parental childcare in many OECD countries need to be lowered if not removed. Support programmes often reduce the costs for low-income families, but out-of-pocket costs often still account for a large share of the earnings for low-paid parents, including single mothers, e.g. in Ireland, the Slovak Republic and the United Kingdom. High childcare costs are one of the factors contributing to inequalities in childcare use across income groups. In European OECD countries, children under the age of three in low-income households are one-third less likely to participate in early childhood education and care (ECEC) than those in high-income households (OECD, 2020a).

Women's incentives to work as a second earner are also influenced heavily by childcare costs. The cost of childcare is particularly critical for single mothers. On average across OECD countries, a low-paid single mother who works full time loses almost two-thirds of her earnings to childcare costs, taxes and the loss of social benefits.

As a consequence, a policy priority for countries should be to step up investment in and increase the supply of affordable, good-quality and safe ECEC so that parents can resume economic activity. But governments should take care to design such measures in a way that preserves equity and boosts parents' work incentives, and introduce suitable price regulations or guidelines to ensure that public support reaches the parents who need it most.

### Parental leave policies for more gender equality

Another measure that countries have been introducing to help families cope with the COVID-19 crisis is emergency parental leave. This was crucial to helping parents at least bridge the impact of lacking childcare and school lockdowns, although the leave periods were not long enough to cover the closure periods. In Italy, for example, working parents with children below 12 were offered 15 days of leave, paid at 50% of the salary, or unpaid leave for parents with children above 12. Alternatively, they were given the choice of taking a voucher to pay for care arrangements.

Again, the pandemic highlights the importance of leave availability and, crucially, parental leave taking for better gender equality. More and more countries now offer paid parental leave, i.e. a longer period of job-protected leave that is shareable between both parents, but usually not as well compensated financially as the maternity or paternity leave that is available after childbirth. A recent trend in an increasing number of OECD countries has been to reserve part of the leave period for fathers or provide fathers with strong incentives to use leave for at least a few months, following the early examples of Iceland and other Nordic countries. By August 2022, according to the European work-life balance directive (Directive (EU) 2019/1158), all EU member states will need to ensure that two of the minimum four months of parental leave are non-transferable between parents.

Parental leave can promote a more equal distribution of unpaid household work in order to avoid that only women experience the related negative effects on their career progression and earnings. But more still needs to happen to ensure that fathers actually take their leave entitlement. In practice, it is mostly mothers who extend their maternity leave with some parental leave, while fathers commonly only take a few days of paternity leave and rarely prolong it. In recent years, however, fathers' uptake of leave has been increasing, for example in Germany, which introduced monetary incentives for fathers to extend and parents to share their leave. Unequal use of parental leave is often rooted in housholds' economic decisions as fathers' leave-taking is often associated with greater income losses than that of mothers due to the gender earning gap. But societal attitudes towards parents' roles and fathers' fears of career implications are also important drivers. There is hope that some fathers at least will increase their participation in unpaid care and non-care work following the experience of the lockdown, but it remains to be seen whether this will be enough to make a lasting change to the substantial gender imbalance.

### Gendered impacts of flexible work and teleworking should not be disregarded

During the pandemic, OECD countries often introduced policies on working time as an adjustment mechanism, allowing for longer working hours, more paid leave and telework. Many countries encouraged the use of teleworking through temporary amendments, independent of whether or how they granted access to telework before the crisis. Telework indeed surged, in particular among female workers (OECD, 2021b). At this time, it is unclear to what extent the increased use of telework is temporary or will persist post-pandemic. It is also unclear whether higher rates of teleworking among female workers reflect gender-related occupational differences and hence potential differences in the "teleworkability" of male and female jobs (Dingel and Neiman, 2020; Boeri and Paccagnella, 2020; Sostero et al., 2020), or rather gender differences in teleworking behaviours *within* occupations.

Gendered changes in work patterns will have direct implications for gender disparities in labour market outcomes, the worklife balance of women and men, and gender equality more broadly. On the positive side, teleworking can support women to stay in employment and in higher-paying full-time jobs after childbirth. By reducing commuting times and increasing schedule control, it is easier to combine family and work responsibilities. It offers an alternative to part-time work, which can relegate women to lower-paying jobs with worse career development opportunities (Chung and van der Horst, 2017). Nonetheless, teleworking also bears risks for gender equality. Workers who use flexible working for care purposes are sometimes seen as less productive or committed. Teleworking women may also be exposed to career penalties, especially if teleworking is not as widespread among men (Chung, 2018).

Finally, there are a range of issues to consider regarding job quality, such as risks of work intensification, degraded work-life balance and blurring boundaries between working and non-working times and spaces (OECD, 2021c). If women telework more often, they will also be more exposed to such risks. Policies and regulations in this area should support an equal, non-penalysing access to (and use of) telework by both women and men.
